# Supplementary material for: Stereotactic Radiosurgery for Lung Cancer with a Risk-Adapted Strategy Using the Volumetric Modulated Arc Therapy Technique: A Single Arm Phase II Study
Source: Cancers (Basel). 2022 Aug 18;14(16):3993. doi: 10.3390/cancers14163993 (PMC9406332; doi:10.3390/cancers14163993)
Supplement: Supplementary file 1 [file cancers-14-03993-s001.zip › cancers-1843791-supplementary.pdf]

Figure S1. Curves of cumulative incidences of local recurrence after stereotactic radiosurgery (SRS), after multi-fraction stereotactic body radiotherapy (SBRT) and previous SBRT data

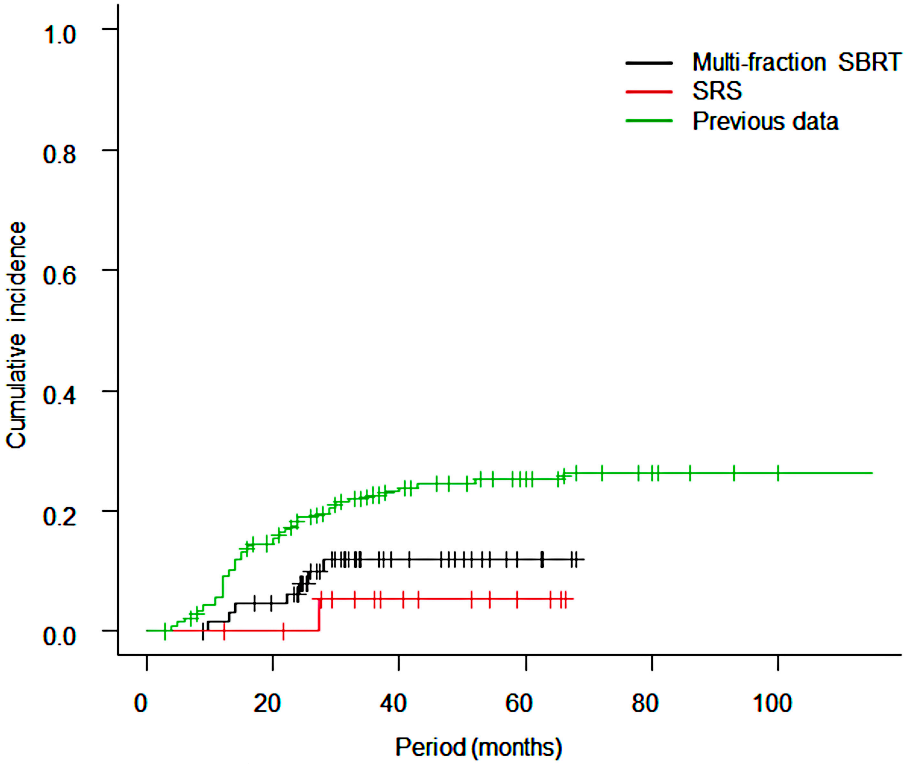

Number at risk

SBRT	66	52	16	4	0	0
SRS	21	18	9	3	0	0
Previous	229	140	67	35	14	5

Table S1. Dose constraints

Organ or tissue	Volume	Volume Max	Max point dose
Lung minus GTV	10%<br>1000 cc	$V_{20\ Gy}$<br>7.4 Gy	
Spinal cord	Any point	10.0 Gy	10.0 Gy
Brachial plexus	3 cc	14.0 Gy	17.5 Gy
Esophagus	5 cc	11.9 Gy	15.4 Gy
Stomach	10 cc	11.2 Gy	12.4 Gy
Heart	15 cc	16.0 Gy	22.0 Gy
Great vessel	10 cc	25.0 Gy	30.0 Gy
Trachea/Main bronchus	4 cc	10.5 Gy	20.2 Gy
Rib	Any point	30.0 Gy	30.0 Gy
Skin	10 cc	23.0 Gy	26.0 Gy

GTV: gross tumor volume

Table S2. Characteristics of patients in the unmatched cohort

	SRS	Multi-fraction SBRT	P value	Standardized difference
Age			0.658	0.122
mean (SD)	72.76 (6.56)	73.76 (9.55)		
Sex			0.199	0.426
Female	3 (14.3%)	21 (31.8%)		
Male	18 (85.7%)	45 (68.2%)		
PS			0.859	0.131
0	9 (42.9%)	30 (45.5%)		
1	10 (47.6%)	32 (48.5%)		
2	2 (9.5%)	4 (6.1%)		
CCI			0.696	0.215
0-1	5 (23.8%)	14 (21.2%)		
2	8 (38.1%)	20 (30.3%)		
3-5	8 (38.1%)	32 (48.5%)		
Diagnosis			0.810	0.174
Pathological diagnosis	9 (42.9%)	28 (42.4%)		
Clinical diagnosis	11 (52.4%)	32 (48.5%)		
Metastasis	1 (4.8%)	6 (9.1%)		
Interstitial shadow			0.133	0.598
Yes	0 (0.0%)	10 (15.2%)		
No	21 (100.0%)	56 (84.8%)		
Tumor diameter, cm			0.155	0.384
mean (SD)	2.01 (0.60)	1.74 (0.78)		

SD: standard deviation, PS: performance status, CCI: Charlson comorbidity index, SRS: stereotactic radiosurgery, SBRT: stereotactic body radiotherapy

Table S3. Patient characteristics of matched cohort

	SRS	Multi-fraction SBRT	P value	Standardized difference
Age			0.478	0.239
mean (SD)	73.83 (5.75)	75.61 (8.81)		
Sex			1.000	0.141
Female	3 (16.7)	4 (22.2)		
Male	15 (83.3)	14 (77.8)		
PS			1.000	<0.001
0	6 (33.3)	6 (33.3)		
1	10 (55.6)	10 (55.6)		
2	2 (11.1)	2 (11.1)		
CCI			0.789	0.231
0-1	4 (22.2)	5 (27.8)		
2	6 (33.3)	7 (38.9)		
3-5	8 (44.4)	6 (33.3)		
Diagnosis			0.943	0.115
Pathological diag	8 (44.4)	9 (50.0)		
Clinical diag	9 (50.0)	8 (44.4)		
Metastasis	1 (5.6)	1 (5.6)		
Interstitial shadow			NA	<0.001
Yes	0 (0.0)	0 (0.0)		
No	18 (100.0)	18 (100.0)		
Tumor diameter, cm			1.000	<0.001
mean (SD)	1.86 (0.48)	1.86 (0.73)		
